# DEAD-Box RNA Helicase DDX47 Maintains Midgut Homeostasis in *Locusta migratoria*

**DOI:** 10.3390/ijms23020586

**Published:** 2022-01-06

**Authors:** Jun-Xiu Wang, En-Bo Ma, Jian-Zhen Zhang, Shu-Ping Xing

**Affiliations:** 1 Research Institute of Applied Biology, Shanxi University, Taiyuan 030006, China; wangjx0419@163.com (J.-X.W.); maenbo2003@sxu.edu.cn (E.-B.M.); zjz@sxu.edu.cn (J.-Z.Z.); 2 College of Life Science, Shanxi University, Taiyuan 030006, China

**Keywords:** DEAD-box RNA helicase, *LmDDX47*, midgut homeostasis, RNAi, *Locusta migratoria*

## Abstract

Tissue homeostasis is critical for maintaining organ shape, size, and function. The condition is regulated by the balance between the generation of new cells and the loss of senescent cells, and it involves many factors and mechanisms. The midgut, an important part of the intestinal tract, is responsible for digestion and nutrient absorption in insects. LmDDX47, the ortholog of DEAD-box helicase 47 from *Locusta migratoria*, is indispensable for sustaining a normal midgut in the nymphs. However, the underlying cellular and molecular mechanisms remain to be elucidated. In this study, *LmDDX47* knockdown resulted in atrophy of the midgut and gastric cecum in both nymph and adult locusts. After *LmDDX47* knockdown, the number of regenerative and columnar cells in the midgut was significantly reduced, and cell death was induced in columnar tissue. LmDDX47 was localized to the nucleolus; this was consistent with the reduction in 18S rRNA synthesis in the *LmDDX47* knockdown group. In addition, the acetylation and crotonylation levels of midgut proteins were significantly increased. Therefore, *LmDDX47* could be a key regulator of midgut homeostasis, regulating 18S rRNA synthesis as well as protein acetylation and crotonylation in the migratory locust.

## 1. Introduction

Tissue homeostasis is required for the morphogenesis and maintenance of organ shape, size, and function [1,2,3,4,5,6,7,8]. Tissue homeostasis is based on a tight balance between the generation of newly differentiated cells and the removal of senescent or damaged cells [1,4,9]. When this balance is disturbed, it results in physical defects [4,10,11]. In insects, this complex coordinated activity is involved in the regulation of programmed cell death, cell proliferation, and differentiation [1,2,3]. It is an essential dynamic process for the survival of insects, which can be affected by both internal and external factors, such as hormones and growth regulators, temperature and other environmental factors [1,2,12,13,14,15].

The intestinal tract, a key organ in the digestive system of insects, is mainly responsible for digestion and nutrient absorption [16,17]. The midgut of insects functionally corresponds to the stomach, small intestine, and colon of mammals. The organ is the primary section for enzyme production and secretion, food digestion, and nutrient absorption [16,17]. In addition, in most insects, the gastric cecum, considered an elongation of the midgut, has been formed as an evolutionary consequence of feeding habits [17,18]. Multiple factors and mechanisms involved in midgut development and homeostasis have been identified in the holometabolous model *Drosophila melanogaster* [3,19,20,21,22,23,24,25]; however, the information in other insects is only fragmented [3,12,19,20,21,22,23,24,25].

The migratory locust *Locusta migratoria* (Orthopetera) is a worldwide agricultural pest that represents a hemimetabolous model. Its midgut, originating from endoderm, consists of three cell types—regenerative cells, differentiated columnar cells, and endocrine cells [1,26]. Regenerative cells are located in the regenerative niche, and they can generate the other two cell types [1]. Differentiated columnar cells are involved in digestive enzyme secretion, digestion, nutrient absorption, and peritrophic membrane formation. These cells are short-lived and are continuously replaced by the progeny of resident regenerative cells [1]. Endocrine cells are scattered among columnar cells and are similar to those in the mammalian intestinal endocrine system [1,27]. To understand the development and maintenance of these cells, the underlying processes and factors must be investigated. Moreover, maintaining a dynamic balance between regenerative and differentiated cells in the midgut of the migratory locust is important for midgut homeostasis.

DEAD-box (DDX) proteins constitute a family of RNA helicases. They are involved in almost every step of RNA metabolism, including transcription, pre-mRNA splicing, ribosome biogenesis, RNA transport, translation initiation, organelle gene expression, and RNA degradation [28,29,30]. DDX47 is conserved from yeast to mammals. In yeast, *Rrp3*, the ortholog of *DDX47*, is required for pre-ribosomal RNA (rRNA) processing. Depletion of *Rrp3* results in processing defects of 35S pre-rRNA and impaired production of 18S rRNA [31]. In addition, DDX47 is a component of the small subunit processome (SSUP) complex, and it influences ribosome biogenesis [32]. SSUP components are key regulators in pluripotent cells. They are preferentially expressed in these stem cells and are critical for regulating global translation [32]. In a tumor cell line, DDX47 interacted with GABA_A_ receptor-associated protein and induced apoptosis and inhibited cell proliferation [33].

In our previous study, we found that *LmDDX47*, the *DDX47* ortholog in *Locusta migratoria*, is essential for midgut maintenance in the third instar nymph [34]. In this study, we further investigated the role of *LmDDX47* in midgut homeostasis. RNAi-mediated *LmDDX47* knockdown resulted in a reduction in the number of regenerative and columnar cells, with the induction of cell death in columnar tissue. Furthermore, 18S rRNA synthesis was reduced in the *LmDDX47* knockdown group, which was consistent with the nucleolar localization of LmDDX47. In addition, the acetylation and crotonylation of midgut proteins were enhanced after the *LmDDX47* knockdown. Therefore, *LmDDX47* is an indispensable regulator required for maintaining a normal midgut, and thus, a potential target in the digestive system for locust control.

## 2. Results

### 2.1. LmDDX47 Knockdown Resulted in Midgut and Gastric Cecum Atrophy in Both Nymph and Adult Locusts

**Figure 1:**
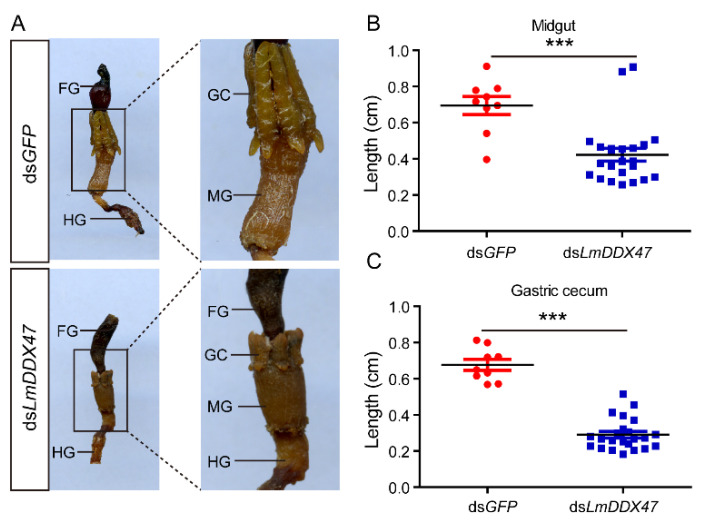
Effects of *LmDDX47* on the intestinal tract of *L. migratoria*. (**A**) *LmDDX47* knockdown led to atrophy of the midgut and gastric cecum. FG: foregut; GC: gastric cecum; MG: midgut; HG: hindgut. (**B**,**C**) Midgut length (**B**) and gastric cecum length (**C**) after *LmDDX47* knockdown. *** *p* < 0.001.

**Figure 2:**
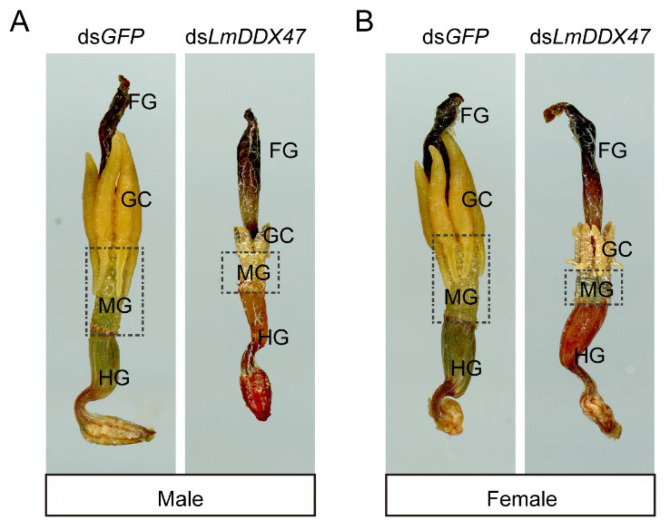
Effects of *LmDDX47* RNAi on the intestinal tract of adult locusts. (**A**,**B**) Intestinal morphology of the adult male (**A**) and female (**B**) migratory locust after *LmDDX47* RNAi. FG: foregut; GC: gastric cecum; MG: midgut; HG: hindgut.

We dissected ds*LmDDX47*-treated fifth instar nymphs 7 days after dsRNA injection and found midgut and gastric cecum atrophy (Figure 1A), a phenotype similar to that observed in third instar locusts [34]. Before ecdysis, the length of the midgut and gastric cecum of double-stranded green fluorescent protein (*GFP*)-treated locusts (control group) was about 7 mm. In the *LmDDX47* knockdown group, the length of the midgut and gastric cecum was reduced to 3 mm (Figure 1B,C). Midgut and gastric cecum atrophy was also found in these ds*LmDDX47*-treated adult locusts (Figure 2).

**Figure 3:**
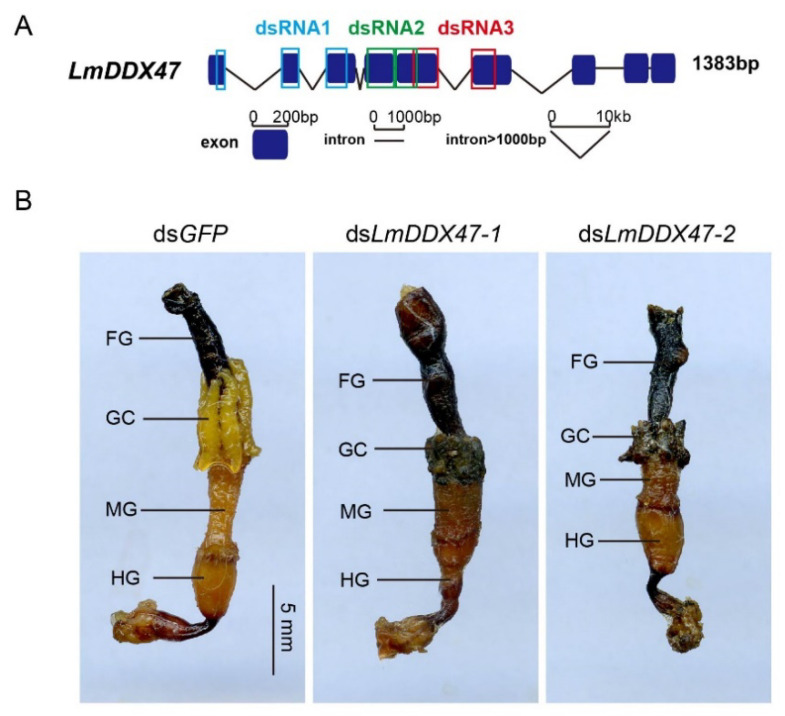
Three corresponding dsRNA regions and phenotypes of *LmDDX47* knockdowns. (**A**) Target regions of three dsRNAs in *LmDDX47* are indicated in different colors. (**B**) Gut phenotypes after injecting ds*LmDDX47*-1 and ds*LmDDX47*-2. FG: foregut; GC: gastric cecum; MG: midgut; HG: hindgut.

To ensure that this phenotype was caused by *LmDDX47* knockdown and was not an off-target effect of RNAi, two additional ds*LmDDX47-1* and *2* were tested, and they produced the same phenotype (Figure 3). Therefore, *LmDDX47* was required for the normal maintenance of the midgut and gastric cecum in both nymph and adult locusts. The following experiments were performed with ds*LmDDX47-3*; thus, ds*LmDDX47* was referred to as ds*LmDDX47-3* other than stated.

### 2.2. Midgut Morphological Changes and Food Consumption after dsLmDDX47 Injection

For an overview of temporal morphological changes in the midgut after ds*LmDDX47* injection, we first evaluated *LmDDX47* expression in the midgut of fifth instar nymphs 24, 48, and 72 h after injecting dsRNA by quantitative real-time PCR (qRT-PCR). *LmDDX47* expression was low at these time points compared with the control locusts injected with the same amount of ds*GFP* (Supplementary Materials Figure S1). This indicates that *LmDDX47* was significantly downregulated from 24 h after dsRNA injection.

**Figure 4:**
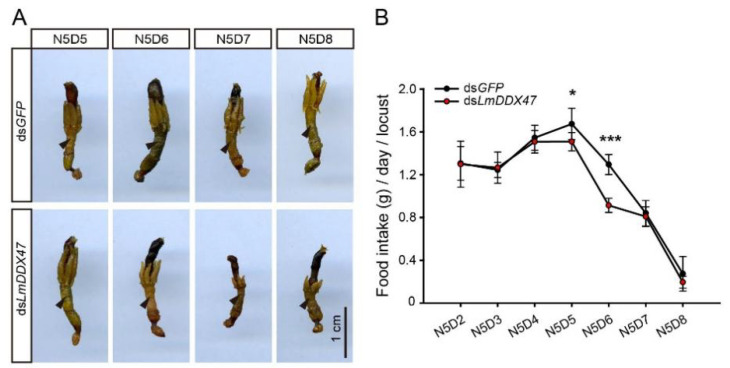
Dynamic changes in intestinal morphology and food intake after *LmDDX47* knockdown. (**A**) Dynamic changes in the intestinal development of *L. migratoria* after *LmDDX47* RNAi. The black arrowhead shows the midgut. Scale bar = 1 cm. (**B**) Average daily food intake (g ± SD) per locust after *LmDDX47* knockdown. * *p* < 0.05; *** *p* < 0.001.

We observed the intestinal morphology on days 4, 5, 6, and 7 after dsRNA injection. On day 4 (N5D5) after *LmDDX47* RNAi, no obvious differences were observed between the ds*LmDDX47*-treated and ds*GFP*-treated groups. On day 5 (N5D6), the midgut and gastric cecum became slightly shorter in ds*LmDDX47*-treated locusts. On day 6 (N5D7), the length of the midgut and gastric cecum was considerably shorter in the ds*LmDDX47*-treated locusts than in the ds*GFP*-treated locusts (Figure 4A). This phenotype remained on day 7 (N5D8).

Except for these morphological changes, food intake significantly decreased in ds*LmDDX47*-treated locusts compared with ds*GFP*-treated locusts. The daily food intake of ds*LmDDX47*-treated locusts was reduced by 11.7% on day 5 and 30.7% on day 6, respectively, compared with the control group (Figure 4B). After day 7, feeding ceased as the locusts entered the preparatory phase for molting into adults. During this period, no significant difference was observed in food intake between the two groups (Figure 4B). Therefore, *LmDDX47* knockdown impaired intestinal functions in the migratory locust and affected its feeding.

### 2.3. Histological Changes in the Intestine of dsLmDDX47-Treated Locusts

**Figure 5:**
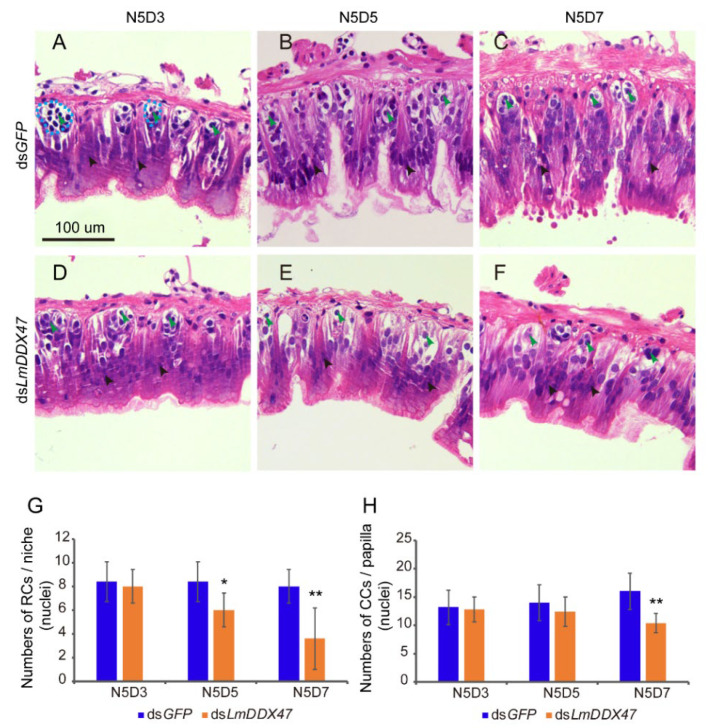
Effect of *LmDDX47* RNAi on the histomorphology of the fifth instar nymph midgut. (**A**–**F**) Sections of the midgut of N5D3, N5D5, and N5D7 nymphs after ds*GFP* and ds*LmDDX47* injections. The blue dotted circle indicates the regeneration niche. Green arrowheads indicate the regenerative cells in the niche, and black arrowheads indicate the columnar cells of the midgut. Scale bar = 100 μm, *n* = 8. (**G**) Number of regenerative cells (nuclei) per niche in the midgut of N5D3, N5D5, and N5D7 nymphs after *LmDDX47* RNAi. (**H**) Number of columnar cells (nuclei) per papilla in the midgut of N5D3, N5D5, and N5D7 nymphs after *LmDDX47* RNAi. RCs: regenerative cells; CCs: columnar cells. * *p* < 0.05; ** *p* < 0.01, *n* = 8.

**Figure 6:**
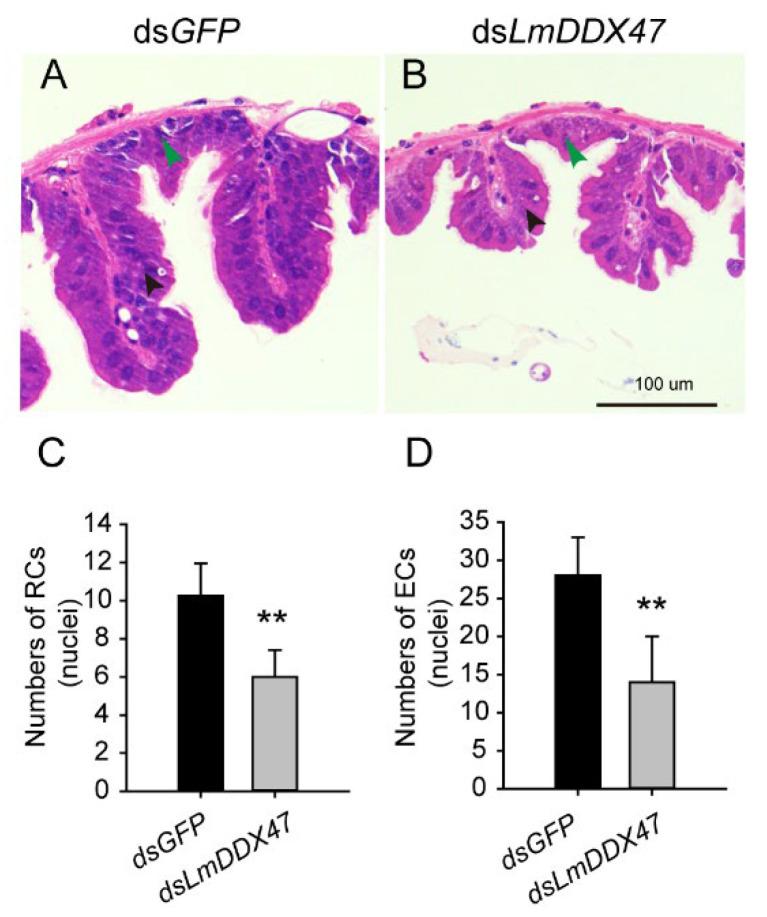
Effects of *LmDDX47* on the gastric cecum of adult locusts. (**A**,**B**) Morphology of the gastric cecum of N5D7 nymphs after *LmDDX47* RNAi. Green arrow heads indicate regenerative cells, and black arrowheads indicate epithelial cells in the gastric cecum. Scale bar = 100 μm, *n* = 5. (**C**,**D**) Number of regenerative and columnar cells (nuclei) in each ridge of the midgut of N5D7 nymphs after *LmDDX47* RNAi. RCs: regenerative cells; ECs: epithelial cells. ** *p* < 0.01, *n* = 5.

To determine the histological changes in the midgut of *LmDDX47* RNAi-treated locusts, the midgut and gastric cecum tissues from fifth instar N5D3, N5D5, and N5D7 nymphs were stained with hematoxylin and eosin (H&E) after dsRNA injection. No significant difference was observed in the number of columnar cells and regenerative cells in N5D3 nymphs between the control and ds*LmDDX47*-treated locusts (Figure 5A,D,G,H). The number of regenerative cells in each niche decreased by 20% in N5D5 nymphs after ds*LmDDX47* treatment (Figure 5B,E,G); however, no obvious difference was observed in the number of columnar cells in each papilla between the two groups (Figure 5B,E,H). The average number of regenerative cells per niche was eight in the control group; however, it decreased to four in N5D7 nymphs of ds*LmDDX47*-treated locusts (Figure 5C,F,G). The number of columnar cells in each papilla decreased by 40% in ds*LmDDX47*-treated locusts compared with ds*GFP*-treated locusts (Figure 5C,F,H). In addition, the ridge of the gastric cecum was significantly shortened, and the number of epithelial and regenerative cells in the gastric cecum was reduced after *LmDDX47* RNAi (Figure 6).

### 2.4. Midgut Columnar Cell Death in dsLmDDX47-Treated Locusts

**Figure 7:**
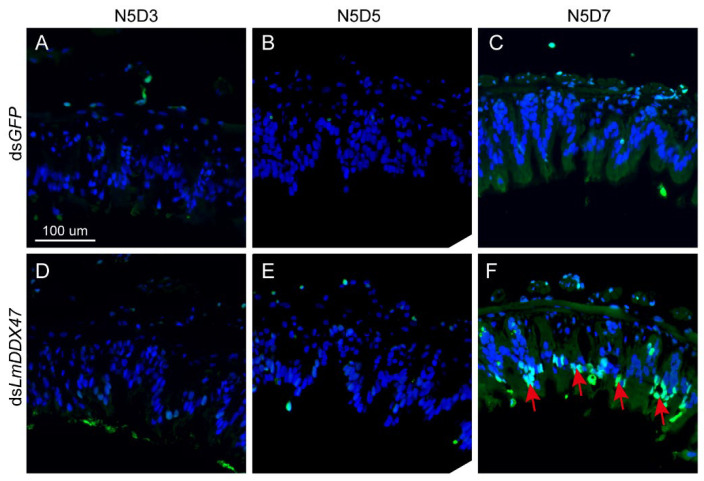
*LmDDX47* knockdown results in cell death in midgut columnar cells. (**A**–**F**) A TUNEL assay of the midgut of N5D3 (**A**,**D**), N5D5 (**B**,**E**), and N5D7 (**C**,**F**) nymphs after ds*GFP* and ds*LmDDX47* injections. Blue represents the nucleus, and red arrows pointing to the green fluorescence indicate the site of cell death in columnar tissue. Scale bar = 100 μm, *n* = 3.

To investigate the cause of the reduction in the number of regenerative and columnar cells in ds*LmDDX47*-treated locusts, we performed terminal deoxynucleotidyl transferase nick-end labeling (TUNEL) staining of the midgut of N5D3, N5D5, and N5D7 nymphs. No obvious cell death signals were observed in the columnar and regenerative cells of the midgut of N5D3 and N5D5 nymphs (Figure 7A,B,D,E). However, strong green signals appeared in the columnar cells of the midgut of N5D7 nymphs after *LmDDX47* knockdown (Figure 7C,F), indicating more cell death occurred in the columnar tissue but not in the regenerative nidi. Because the TUNEL assay cannot directly tell apoptosis from necrosis [35], further investigation is still required to determine what type of cell death happened in the columnar tissue. Nevertheless, the advanced cell death contributed to a partial reduction in the number of columnar cells. However, it played no role in reducing the number of regenerative cells.

### 2.5. LmDDX47 Is Localized to the Nucleolus, and LmDDX47 Knockdown Affected 18S rRNA Synthesis

**Figure 8:**
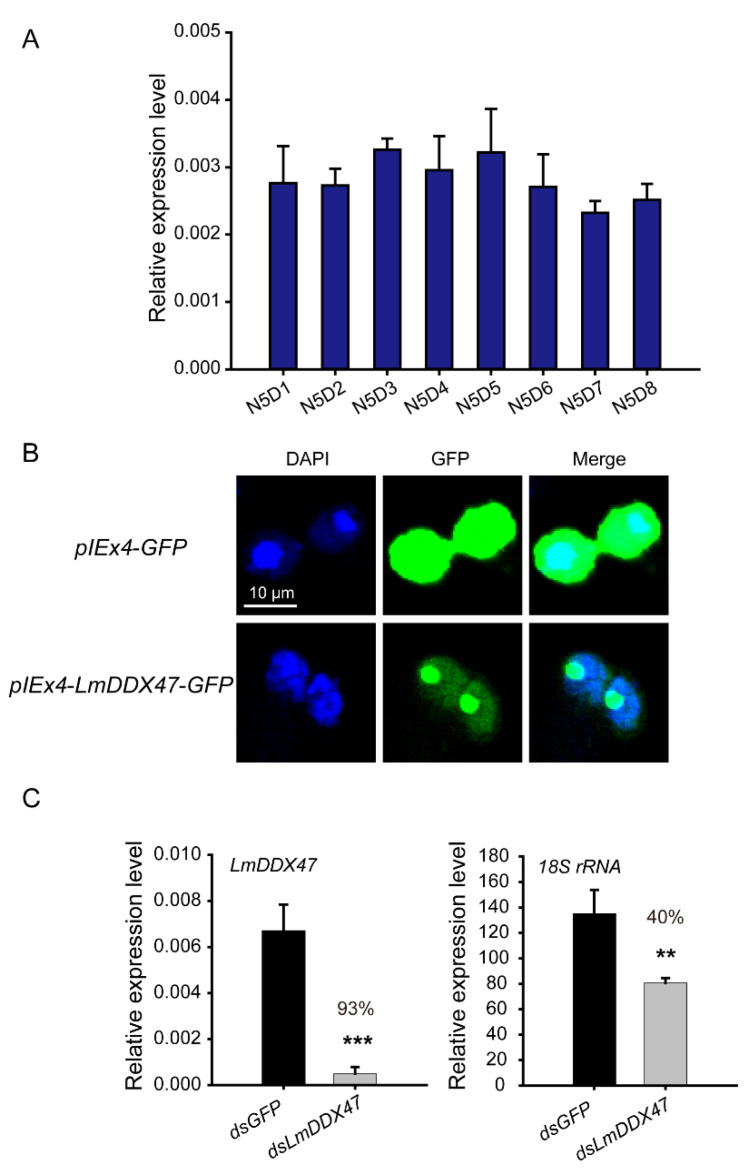
Relative expression of *LmDDX47* in the midgut of fifth instar nymphs and its subcellular localization. (**A**) Relative expression of *LmDDX47* in the midgut of fifth instar nymphs (N5D1–N5D8) by qRT-PCR. *RPL32* was used as the reference gene. All data are presented as mean ± SD of five independent biological replicates. (**B**) Subcellular localization of LmDDX47 detected by transient transfection with the *pIEx4*-*LmDDX47*-*GFP* construct in *Drosophila* S2 cells. Transfection with the *pIEx4*-*GFP* vector was the control. Blue indicates the nucleus, and green represents GFP expression. (**C**) Expression of *18S rRNA* and *LmDDX47* was detected in the midgut of N5D7 nymphs after *LmDDX47* RNAi. *RPL32* was used as the reference gene. All data are presented as means ± SD of six independent biological replicates. ** *p* < 0.01; *** *p* < 0.001.

*LmDDX47* is expressed in the midgut of locusts. To elucidate its function, we used qRT-PCR to assess the dynamic changes of *LmDDX47* mRNA levels in the midgut of fifth instar nymphs. *LmDDX47* transcript levels were relatively low, but *LmDDX47* was constantly expressed during the fifth instar nymph stage (Figure 8A). Analysis of S2 cells transiently transfected with the *pIEx4*-*LmDDX47*-*GFP* construct (Figure 8B) indicated that LmDDX47 was localized to the nucleolus, the site of rRNA synthesis and ribosome assembly. This suggested that LmDDX47 was involved in rRNA synthesis. Using qRT-PCR, we assessed 18S rRNA expression, which decreased by 40% after *LmDDX47* RNAi (Figure 8C). Therefore, *LmDDX47* influenced rRNA synthesis.

### 2.6. Effects of LmDDX47 RNAi on Protein Modifications in the Locust Midgut

**Figure 9:**
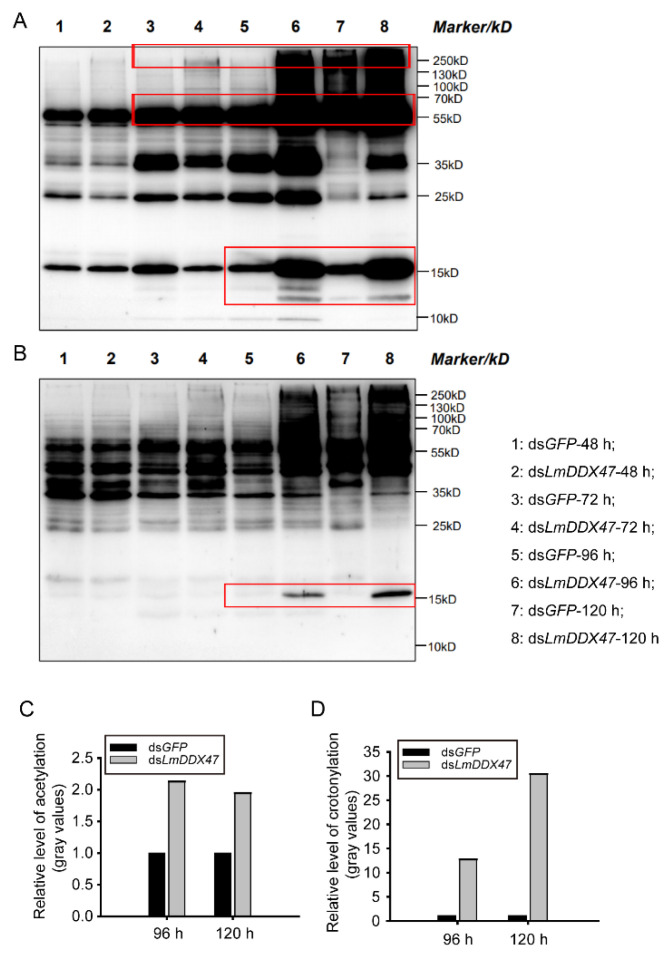
Effects of *LmDDX47* on protein modifications in the locust midgut. (**A**,**B**) Acetylation (**A**) and crotonylation (**B**) levels of midgut proteins were estimated using Western blotting at 48, 72, 96, and 120 h after *LmDDX47* RNAi. The red box indicates that the modification of the sample proteins had significantly increased in the *LmDDX47* RNAi group. (**C**,**D**) Relative gray values of acetylation (**C**) and crotonylation (**D**) levels in the 15 kD protein in the ds*LmDDX47*-treated group compared with the ds*GFP*-treated group.

To clarify the molecular mechanisms underlying the role of *LmDDX47* in midgut homeostasis, we conducted RNA sequencing (RNA-seq) on samples from the midgut at 48 h after ds*LmDDX47* injection. In total, 32 up-regulated and 109 down-regulated genes were obtained (Tables S2 and S3). Six up-regulated and twelve down-regulated genes (fold change > 1.5) were selected to do validation. Unfortunately, none was confirmed with significant expression differences (Supplementary Materials Figures S2 and S3). Moreover, we analyzed several genes in the apoptosis pathway by qRT-PCR; no significant differences were observed between the transcriptional levels of these genes in the ds*GFP*-treated and ds*LmDDX47*-treated locusts (Supplementary Materials Figure S4). In this study, we focused on the effect of *LmDDX47* on the post-translational modifications of midgut proteins. We assessed the acetylation and crotonylation levels of midgut proteins at 48, 72, 96, and 120 h after ds*LmDDX47* injection. Protein bands were clear and uniform, without protein degradation (Supplementary Materials Figure S5). No differences were observed in protein modification levels at 48 h (Figure 9). However, after 96 h, the acetylation level of 10–15, 55–70, and 250 kD proteins increased significantly (such as the 15 kD protein), and it was nearly twice that in the control group (Figure 9A,C). The crotonylation level of the 15 kD protein increased 12.7 and 30.4 folds following *LmDDX47* RNAi after 96 and 120 h, respectively, compared with the control group (Figure 9B,D). Therefore, *LmDDX47* knockdown resulted in the undesired modification of proteins, which functioned in the midgut, leading to physical and physiological defects.

## 3. Discussion

*LmDDX47* knockdown resulted in serious defects in the midgut and gastric cecum of both nymph and adult locusts, suggesting that *LmDDX47* was a key regulator in midgut homeostasis and that it was required for maintaining normal midgut development during the various stages of the insect life cycle. DDX47 is highly conserved in insects and mammals [34]. In humans, DDX47 could be indispensable to normal intestinal development, and its absence could result in abnormal intestinal development and related disorders. Therefore, exploring the function of DDX47 in other species is essential.

The number of regenerative cells was significantly reduced in the midgut and gastric cecum of locusts after ds*LmDDX47* injection. DDX47 is a component of the SSUP complex and is important for controlling translation in stem cells [32]. *LmDDX47* knockdown could partially inhibit the generation and renewal of regenerative cells. In addition, the number of columnar cells decreased in ds*LmDDX47*-treated locusts, which could be attributed to advanced cell death in the tissue. In *Drosophila*, the balance between the rate of generation of intestinal stem cells and the rate of degeneration of senescent cells is critical for gut development. This balance is regulated by many factors and mechanisms such as the Hippo, JNK, Cytokine/JAK/STAT, EGFR/Ras/Raf, and JAK/STAT pathways, and cellular redox state [5,10,20,21,23,36,37,38]. Excessive injury of epithelial cells or inhibition of the regeneration and differentiation of intestinal stem cells causes cellular imbalances, resulting in the disruption of homeostasis and intestinal developmental defects [4,10,11]. Therefore, to maintain midgut homeostasis, we speculate that *LmDDX47* is required for maintaining self-renewal in regenerative cells and inhibiting cell death in columnar cells. However, the mechanisms underlying *LmDDX47* function in *L. migratoria* are required to be elucidated.

DDX47 is located in the nucleolus of hamster BHK21 cells and is involved in 18S rRNA synthesis in both yeast and *Arabidopsis* [31,39,40]. In line with these previous studies, LmDDX47 was also localized to the nucleolus, and 18S rRNA expression was significantly reduced after *LmDDX47* knockdown. In *Drosophila*, rRNA synthesis is regulated by *dMyc*, which is required for intestinal stem cell proliferation and maintenance; it acts downstream of the Hippo, JAK/STAT, and EGFR pathways [41,42]. Therefore, the reduction in 18S rRNA synthesis contributed to midgut defects, at least partially, in *L. migratoria*.

At the transcriptome level, RNA-Seq did not yield any direct target genes for *LmDDX47*, and no significant differences were observed in the expression of apoptosis pathway genes between the ds*LmDDX47*-treated and ds*GFP*-treated locusts, suggesting that *LmDDX47* had no effect on the transcription of most genes. Considering post-translational modifications, the acetylation and crotonylation levels of midgut proteins increased after ds*LmDDX47* treatment. Therefore, *LmDDX47* regulates the post-translational modifications of midgut proteins. In eukaryotic cells, acetylated proteins perform an important role in the epigenetic regulation of gene expression and metabolic regulation [43,44]. Lysine acetylation and crotonylation are involved in diverse cellular processes such as chromatin remodeling, cell cycle, splicing, nuclear transport, and cellular organization [45,46]. Further studies are necessary to identify the abnormally modified proteins and to understand whether these modifications inhibit intestinal development and control midgut homeostasis in the migratory locust.

In summary, *LmDDX47* plays an important role in maintaining midgut homeostasis in *L. migratoria* by regulating 18S rRNA synthesis and modifying proteins to maintain the balance between the number of regenerative and columnar cells. The data from this study provide only some fundamental information, so further investigation is still required for understanding the molecular mechanism of *LmDDX47* in the maintenance of a normal midgut in locusts.

## 4. Materials and Methods

### 4.1. Insect Rearing and Cell Lines

*Locusta migratoria* eggs were purchased from a locust breeding center in Hebei, China, and maintained at 30 ± 2 °C, 50% relative humidity, and a 14:10 h (light:dark) photoperiod for hatching. Locust nymphs were maintained under the same conditions. They were fed with fresh wheat leaves twice a day. Fifth instar nymphs and adult locusts will be used for RNA interference analyses.

The *Drosophila* S2 cell line was derived from a primary culture of *Drosophila melanogaster* embryos in the late stage (20–24 h old). *Drosophila* S2 cells were obtained from the Institute of Applied Biology, Shanxi University. S2 cells were cultured in Schneider’s *Drosophila* medium (Thermo Fisher Scientific, Shanghai, China) supplemented with 10% fetal bovine serum. Cells were incubated at 27 °C.

### 4.2. pIEx4-LmDDX47-GFP Construction and LmDDX47 Subcellular Localization

The *pIEx4-GFP* vector was obtained from the Institute of Applied Biology, Shanxi University. *LmDDX47* was inserted into the *pIEx4-GFP* vector at the HpaI and SacI restriction sites. The vector primer details are provided in Table S1. The *pIEx4*-*LmDDX47*-*GFP* construct was verified by sequencing and transfected into S2 cells. Transfected S2 cells were cultured for 48 h, stained with 4′,6-diamidino-2-phenylindole (DAPI), and observed for GFP expression using an LSM 880 confocal microscope (Zeiss, Jena, Germany).

### 4.3. RNA Extraction and qRT-PCR Analysis

The midgut of fifth instar nymphs (N5D1–N5D8) was used to analyze the developmental expression of *LmDDX47*. Total RNA was extracted using RNAiso Plus (TaKaRa, Tokyo, Japan) according to the manufacturer’s protocol. Three nymphs were used in each replicate, and five independent biological replicates were performed for each stage. The quality and quantity of total RNA were assessed by 1.5% agarose gel electrophoresis and a NanoDrop 2000 spectrophotometer (Thermo Fisher Scientific, Waltham, MA, USA). cDNA synthesis, qRT-PCR analysis, and quantification of relative mRNA levels of target genes were performed as described previously [34]. *RPL32* was used as the internal reference gene. Primer details are provided in Table S1.

### 4.4. RNA Interference of LmDDX47

To understand the role of *LmDDX47* in midgut development, three dsRNAs corresponding to different regions of *LmDDX47* were synthesized [34]. The specific primer details are listed in Table S1. Approximately 7.5 μg and 10 μg of ds*LmDDX47* were injected into the abdomen of fifth instar nymphs (N5D1, molting within 12 h) and adults, respectively, using a manual microinjector (Gaoge, Shanghai, China). ds*GFP* was used as the negative control. Three biological replicates were used for dsRNA injection, with 15 nymphs in each replicate. To test the silencing efficiency, the *LmDDX47* mRNA level was analyzed by qRT-PCR at 24, 48, and 72 h after dsRNA injection. The remaining nymphs were maintained under the same conditions to observe their phenotypes.

### 4.5. Measurement of Food Intake

Locusts were provided sufficient wheat leaves every day. The average daily food consumption of each locust was calculated based on the difference between the daily food supply and residual portions. Five biological replicates with five locusts in each replicate were evaluated.

### 4.6. Histological Analysis and Cell Number Quantification

To investigate the effect of *LmDDX47* knockdown on cells of the midgut and gastric cecum, paraffin sections were prepared and stained with H&E. The midgut and gastric cecum were collected from fifth instar N5D3, N5D5, and N5D7 nymphs treated with either ds*LmDDX47* or ds*GFP*. Tissues were fixed using 2.5% glutaraldehyde, washed, dehydrated, made transparent, treated with wax, and embedded in paraffin to obtain tissue sections. Sections of 5 μm were stained with H&E as described previously [47]. Images were recorded under a fluorescence microscope (Olympus BX51, Tokyo, Japan).

For the quantification of the cell number per regenerative niche or per columnar papilla in the section of the midgut, eight different sections were observed. Eight regenerative niches and five columnar papillae per section were randomly selected for cell number counting.

### 4.7. TUNEL Staining

Paraffin sections of the midgut were dewaxed, permeated with proteinase K, and labeled with FITC-12-dUTP using the Fluorescein Tunel Cell Apoptosis Detection Kit (Servicebio Technology, Wuhan, China) according to the manufacturer’s instructions. The sections were stained with DAPI and photographed with an LSM 880 confocal microscope (Zeiss, Jena, Germany).

### 4.8. Western Blotting

The Western blot was performed in PTM Biolabs Inc., Hangzhou, China. In brief, midgut proteins were extracted at 48, 72, 96, and 120 h after ds*GFP* and ds*LmDDX47* injections. Proteins (15 μg) were separated using a 12% SDS-PAGE gel and transferred to a polyvinylidene difluoride membrane. The membrane was blocked with 5% milk and incubated with anti-acetyl lysine antibody (1:1000; PTM-101, PTM Biolabs Inc., Hangzhou, China) or anti-crotonyl lysine antibody (1:1000; PTM-502, PTM Biolabs Inc.). Goat anti-mouse IgG (H + L) (1:10000; Thermo Fisher Scientific) was used as the secondary antibody. Images were captured using a gel imaging instrument. The procedure was performed according to a previous method [48]. Relative gray values were evaluated with ImageJ software.

### 4.9. Statistical Analysis

*LmDDX47* expression in the midgut of fifth instar nymphs on different days was compared by one-way analysis of variance (ANOVA) and Tukey’s test. The remaining data were evaluated by Student’s *t*-test. Significant differences between the results of the control and treatment groups are marked with asterisks (* *p* < 0.05; ** *p* < 0.01; *** *p* < 0.001). All data are presented as means ± SD. Each experiment was repeated at least thrice.

### 4.10. RNA-Sequencing and Analysis

Fifth instar nymphs at N5D1 were injected with 7.5 μg ds*GFP* and ds*LmDDX47*, respectively. At 48 h after injection, the midguts were sampled for total RNA extraction. Two biological replicates were repeated with six locusts in each replicate. The preparation of complementary DNA (cDNA) libraries and RNA-sequencing (RNA-seq) was performed by Biomarker (Biomarker Technologies, Beijing, China) [49]. Differentially expressed genes (DEGs) from the RNA-seq data were analyzed using DESeq2 based on the criteria of FDR (false discovery rate) > 1.5. qRT-PCR on different RNA templates was performed to validate the DEGs. Primers used here are listed in Table S1.

## Data Availability

Data are contained within the article or Supplementary Materials.

## Supplementary Materials

The following supporting information can be downloaded at: https://www.mdpi.com/article/10.3390/ijms23020586/s1.
