# Supplementary material for: DEAD-Box RNA Helicase DDX47 Maintains Midgut Homeostasis in Locusta migratoria
Source: Int J Mol Sci. 2022 Jan 6;23(2):586. doi: 10.3390/ijms23020586 (PMC8775783; doi:10.3390/ijms23020586)
Supplement: Supplementary file 1 [file ijms-23-00586-s001.zip › ijms-1483323-supplementary.pdf]

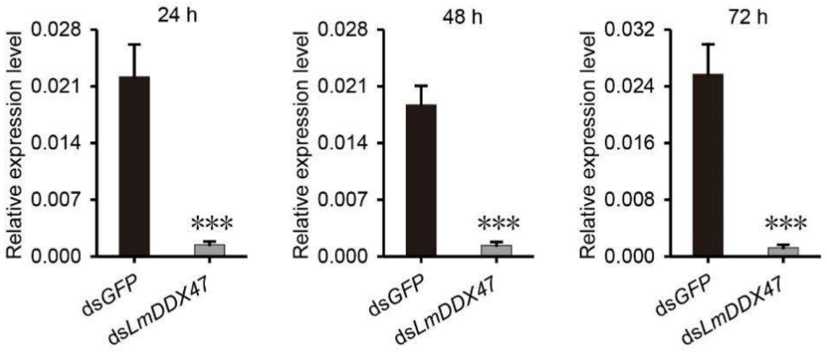

Figure S1. *LmDDX47* mRNA expression level at 24, 48, and 72 h after treatment with ds*GFP* and ds*LmDDX47* was detected by qRT-PCR. $n = 6$. ***, $p < 0.001$.

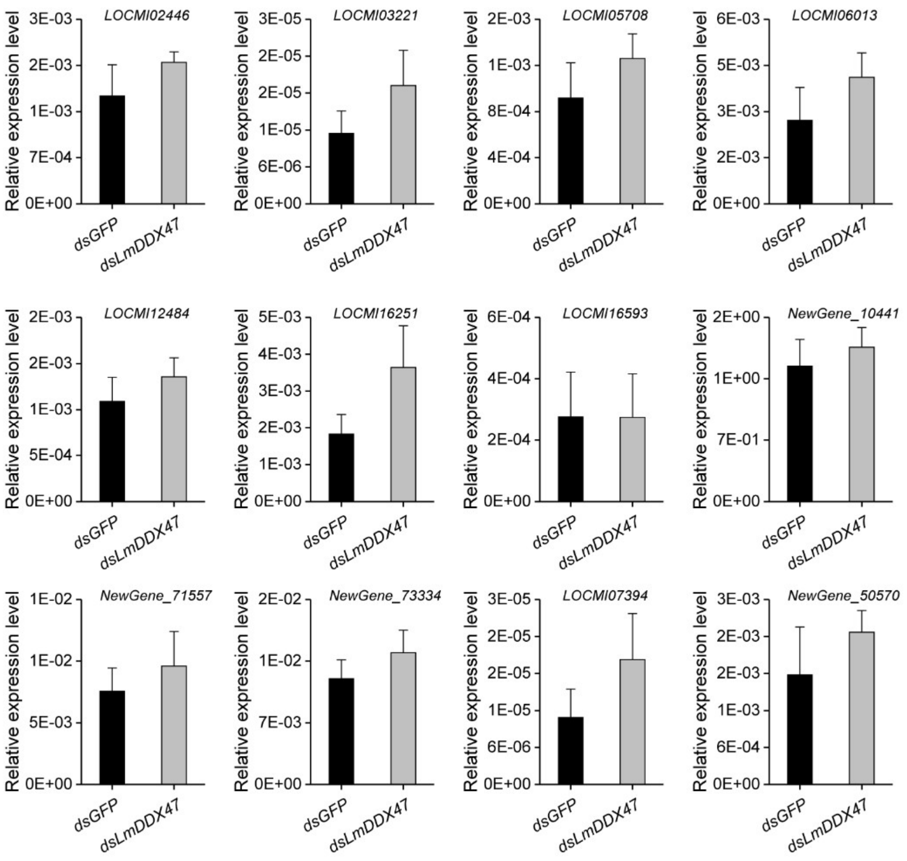

Figure S2. Validation of 12 down-regulated expression genes from RNA-seq data by qRT-PCR. *RPL32* was used as the reference gene. The data are presented as means ± SD of three independent biological replicates. No significant difference was observed between the groups.

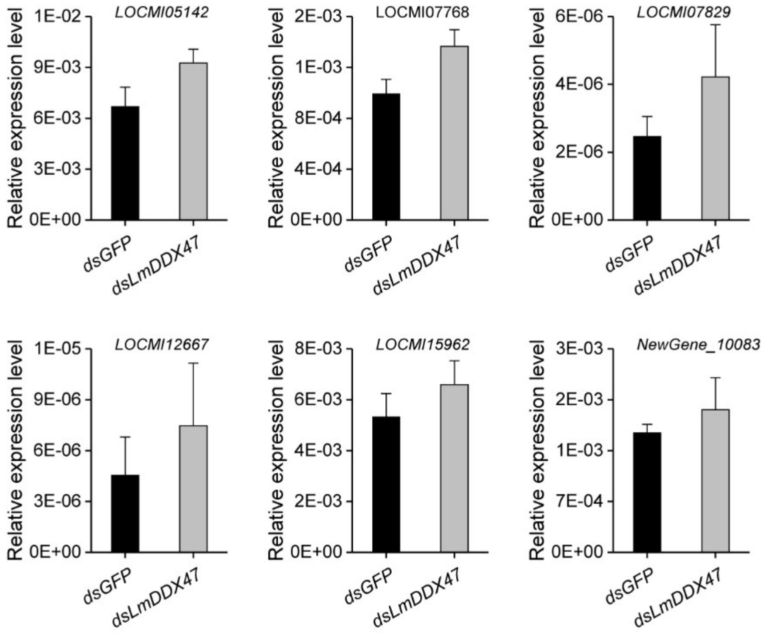

Figure S3. Validation of 6 up-regulated expression genes from RNA-seq data by qRT-PCR. *RPL32* was used as the reference gene. The data are presented as means ± SD of three independent biological replicates. No significant difference was observed between the groups.

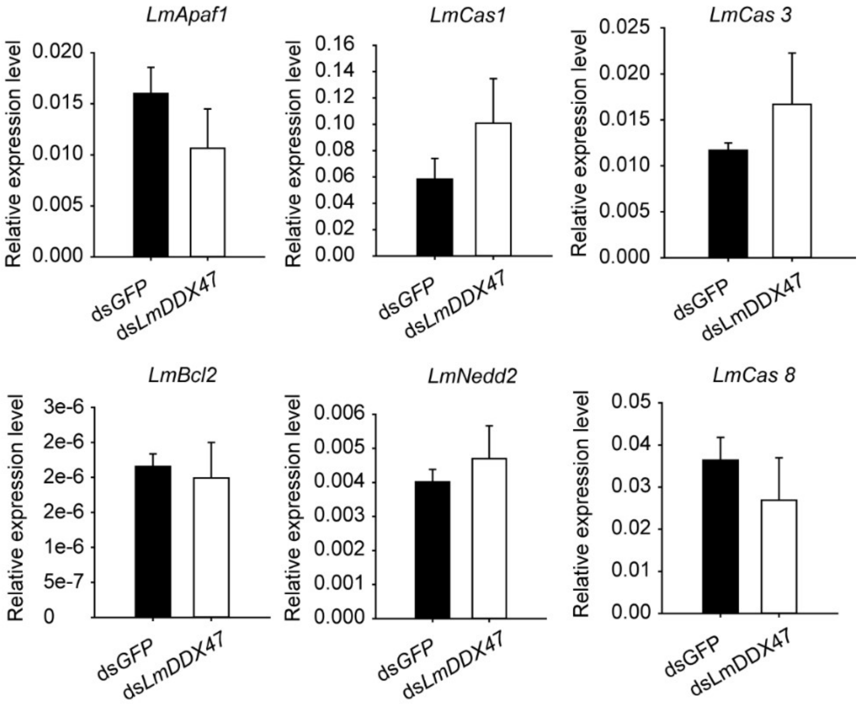

Figure S4. Effect of *LmDDX47* RNAi on the expression of selected genes in the apoptosis pathway. Expression of *LmApaf1*, *LmCas1*, *LmCas 3*, *LmBcl2*, *LmNedd2*, and *LmCas 8* was detected in the midgut of N5D7 nymphs after *LmDDX47* knockdown. *RPL32* was used as the reference gene. All data are presented as means ± SD of six independent biological replicates. No significant difference was observed between the groups.

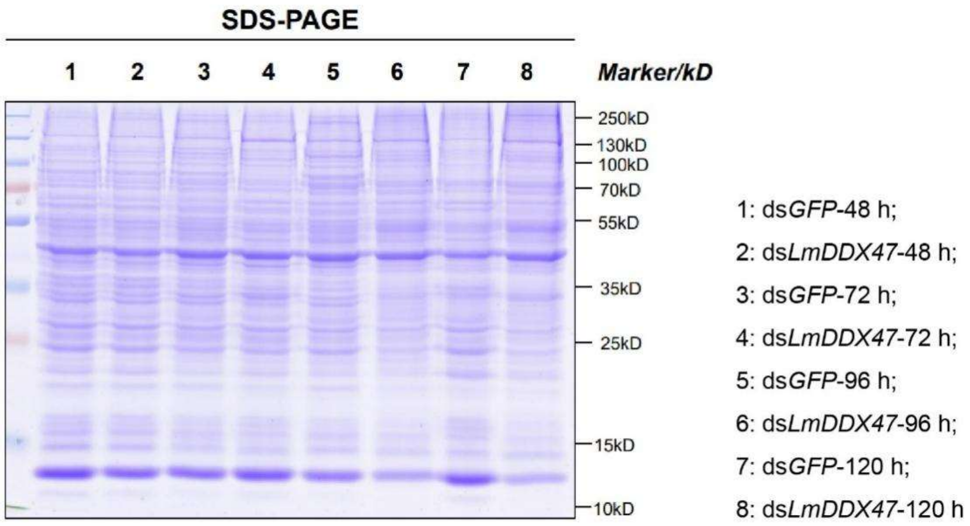

Figure S5. Midgut protein level was estimated by 12% SDS-PAGE at 48, 72, 96, and 120 h after *LmDDX47* RNAi.
