# Supplementary material for: DEAD-Box RNA Helicase DDX47 Maintains Midgut Homeostasis in Locusta migratoria
Source: Int J Mol Sci. 2022 Jan 6;23(2):586. doi: 10.3390/ijms23020586 (PMC8775783; doi:10.3390/ijms23020586)
Supplement: Supplementary file 1 [file ijms-23-00586-s001.zip › Table S1.pdf]

Table S1 Primer sequences used in this study

Primer list	Primer sequence (5'-3')	Application
GFP-RNAi-F	taatacgactcactatagggGACGTAAACGGCCACAAGTT	Synthetic dsRNA
GFP-RNAi-R	taatacgactcactatagggTGTTCTGCTGGTAGTGGTCG	
LmDDX47-RNAi-F1	taatacgactcactatagggTTGTTGAAGAGGACAATGAGA	Synthetic dsRNA1
LmDDX47-RNAi-R1	taatacgactcactatagggGTAAGTATGAGAGCAAAATATC	
LmDDX47-RNAi-F2	taatacgactcactatagggTGCTGTGATTGTTGGTGGTA	Synthetic dsRNA2
LmDDX47-RNAi-R2	taatacgactcactatagggTTCTTTGTCATTGTGGCTGA	
LmDDX47-RNAi-F3	taatacgactcactatagggAGCCACAATGACAAAGAA	Synthetic dsRNA 3
LmDDX47-RNAi-R3	taatacgactcactatagggGTAGAGGCACAGCGGTAA	
LmDDX47-RT-F	ATGGCAAGTGATAATAATATG	RT-qPCR
LmDDX47-RT-R	AAGTTGCTCACATGCTTCAC	
Lm18S rRNA-RT-F	CCTGCGGCTTAATTTGACTC	RT-qPCR
Lm18S rRNA-RT-R	GTAATCGCTGACAGCACGAA	
LmApaf-RT-F	ACAGAGGAAGGTGGAATAAC	RT-qPCR
LmApaf-RT-R	TGGTAGATTTCCCACAGACA	
LmCas1-RT-F	TTGCGAACAGAAACAGACG	RT-qPCR
LmCas1-RT-R	CAAAGGGCTTGCATGAACC	
LmCas3-RT-F	ACAATCAGCCAAAGCCATTC	RT-qPCR
LmCas3-RT-R	AGGCCCCTTTTCTTGTGAGT	
LmBcl2-RT-F	AAGTCCGCAGGGCTCATC	RT-qPCR
LmBcl2-RT-R	ATGTAGTCGGCGTGGTTCT	
LmNedd2-RT-F	GGTTACGGGATGTTGAGTGG	RT-qPCR
LmNedd2-RT-R	CCTTATGTGGAGGTGGAGAT	
LmCas8-RT-F	CGATTGGGCTAGAGTGGAAA	RT-qPCR
LmCas8-RT-R	TTGCCACTATGATTGGTGGA	
LmDDX47-HpaI-F	TGCGgttaacATGGCAAGTGATAATAATATGG	Constructed pIEx4-*LmDDX47-GFP*
LmDDX47-SacI-R	TATCgagctccTCTGTGTTTCTTCATCTTCTTC	
LOCMI02446- RT-S	GAGTGAAGCAATGGGTCCTGAT	RT-qPCR
LOCMI02446- RT-A	TGATTTGTCCATACTGCCACCA	
LOCMI03221- RT-S	CCGTGGAGTGAAGAGGAGGG	
LOCMI03221- RT-A	GGATGAACTCTGGCTTGGCTTT	
LOCMI05708- RT-S	CCCTGTGCTTGCGTCTATAATGT	
LOCMI05708 -RT-A	GTAGCGATTCCCAGCCTCCAGAT	

LOCMI06013- RT-S	TTCGAGGTAACTTCAGTAGACGC
LOCMI06013- RT-A	TCCAAGGCAAATCACAGCATCA
LOCMI07394- RT-S	GTGCTGGCATCGGACTACCTCA
LOCMI07394- RT-A	CACCCATCATTATCCTCAACACCTTC
LOCMI12484- RT-S	AACATTTGGACCTGTGATATTTGC
LOCMI12484 RT--A	GTTGGGTCTTCTTCCTGATTGC
LOCMI15993- RT-S	GGTGCTTGGTTCTGTTGGTG
LOCMI15993- RT-A	GATCCTGGTTTCATGGCTGT
LOCMI16251- RT-S	CTGGGAATACGGACTGGAAGT
LOCMI16251- RT-A	GTAGTTGGTGGCAGTGAGGC
L OCMI116593- RT-S	TGATGGGCTACAACGAGATATGCG
L OCMI116593- RT-A	TGCGACTTGAGCCTGATGGACT
NewGene10441- RT-S	GTAAAGCAGTCAACAATCCGAAGA
NewGene10441- RT-A	TGGCTGGGTAAGTCAGTTCG
NewGene50570- RT-S	TACTCCATCCAGGTCACAGCAGC
NewGene50570- RT-A	CAATTCGGAATGTCGTTTGTTTCT
NewGene71557- RT-S	CGGCTCATTGGACTTCTCAG
NewGene71557- RT-A	GCCCAGCAGTAGTATTCTTCC
NewGene73334- RT-S	TGACCAGCGTCTTACCTTGTTAC
NewGene73334- RT-A	ACCTTGTTCTCCGTGATCCTTTA
LOCMI05142- RT-S	TTAGCAACAGATGGCGATTA
LOCMI05142- RT-A	CTCCAGCATACAGCAGACAC
LOCMI07768- RT-S	TAGAGGAGTACCCAGATTGC
LOCMI07768- RT-A	CACTGTGCCTTTCAGATGTA
LOCMI07829- RT-S	GTTTTCCTTCATCAGGTCTT
LOCMI07829- RT-A	AATATCATGCTGCTGTTGTA
LOCMI2667- RT-S	GTGCGGCTGCTGCGGATGGA
LOCMI2667- RT-A	GAGGGCTGCGTGCTGTGGTAG
LOCMI15962- RT-S	GACAGGGTTGTGCCGTCTTC
LOCMI15962- RT-A	TCTTGCCCGCTTCTTGCTTA
NewGene10083- RT-S	GGAATAACGCCTGTTGAGGGTAC
NewGene10083- RT-A	AACGCTGAATATGACATGACTTGCA
